# Mapping High-Level Evidence in Neuroanesthesia: A Scoping Review of Multicenter Randomized Controlled Trials in Anesthesia for Neurosurgery

**DOI:** 10.3390/jcm15052012

**Published:** 2026-03-06

**Authors:** Mouad Elganga, Abramo Aziz Rizk, Tumul Chowdhury

**Affiliations:** 1 Temerty Faculty of Medicine, University of Toronto, Toronto, ON M5S 3K3, Canada; mouad.elganga@mail.utoronto.ca; 2 Faculty of Health Sciences, McMaster University, Hamilton, ON L8S 4L8, Canada; abramorizk@gmail.com; 3 Department of Anesthesiology and Perioperative Medicine, Marnix E. Heersink School of Medicine, The University of Alabama at Birmingham, Birmingham, AL 35233, USA; 4 Department of Neurosurgery, Marnix E. Heersink School of Medicine, The University of Alabama at Birmingham, Birmingham, AL 35233, USA

**Keywords:** neuroanesthesia, neurosurgery, perioperative care, multicenter randomized controlled trials, scoping review

## Abstract

**Background/Objectives**: Anesthesia for intracranial neurosurgery presents unique challenges because of the sensitivity of the brain to perioperative physiological disturbances, yet neuroanesthetic practice remains highly variable and supported by a limited high-level evidence base. We conducted a scoping review to map and characterize multicenter randomized controlled trials (RCTs) evaluating perioperative management strategies in adults undergoing intracranial neurosurgery. **Methods**: This scoping review was reported in accordance with the PRISMA extension for Scoping Reviews. MEDLINE, PubMed, EMBASE, Cochrane Central, and Web of Science were searched from inception to 25 June 2025. Multicenter RCTs enrolling adults undergoing intracranial neurosurgery and evaluating anesthetic, hemodynamic, ventilatory, or perioperative interventions were included. We prioritized mapping multicenter designs for their greater external validity and implementation potential. Data were extracted in duplicate and summarized descriptively. **Results**: Of 417 records identified, 13 multicenter trials (≥2 recruiting sites) involving 2765 participants across nine countries from 1997–2025 were included. Most trials evaluated anesthetic maintenance or opioid regimens (7/13), followed by post-craniotomy pain control (3/13), ventilation/brain relaxation strategies (1/13), antiemetic prophylaxis (1/13), and temperature management (1/13). Outcomes were predominantly short-term and process-based (hemodynamics 7/13, opioid use 7/13, emergence metrics 5/13). Patient-centered outcomes were rarely measured (mortality 1/13, functional neurological outcome 1/13, cognitive outcome 1/13; quality of life 0/13). Only one trial assessed outcomes at ≥72 h postoperatively. Over half of the included trials were judged at high risk of bias. **Conclusions**: Multicenter RCT activity in neuroanesthesia remains sparse and narrowly focused, highlighting the need for larger, methodologically robust trials targeting patient-centered and long-term outcomes.

## 1. Introduction

Neurosurgical procedures represent some of the most physiologically demanding contexts in anesthetic practice. The administration of anesthesia for intracranial surgery requires continuous optimization of cerebral hemodynamics, intracranial pressure, and cerebral metabolic demands to maintain neuronal integrity and facilitate favorable surgical conditions. Even brief perturbations in mean arterial pressure, carbon dioxide tension, or cerebral perfusion pressure can result in cerebral ischemia, edema, or irreversible neurological injury [1]. Patients undergoing intracranial neurosurgery are also at substantial risk of postoperative neurocognitive complications; postoperative delirium alone occurs in approximately 12–26% of patients and is associated with prolonged recovery and worse functional outcomes [2].

However, despite significant advances in anesthetic, hemodynamic, and neuromonitoring techniques, perioperative practices in the neurosurgical domain remain highly variable across institutions and often rely on local expertise rather than high-level evidence. In the absence of definitive research and consensus guidelines, anesthesiologists frequently must base neuroanesthetic management on institutional culture or personal experience. This variability reflects a broader paucity of high-quality, standardized evidence in neuroanesthesia.

There is growing recognition that rigorous clinical research is needed to inform and harmonize neuroanesthetic care. Randomized controlled trials (RCTs) are among the strongest study designs for evaluating causal effects of perioperative interventions; however, single-center trials often struggle with limited sample sizes, narrow patient populations, and center-specific practices that restrict external validity [3]. Multicenter RCTs can address some of these limitations by enrolling more diverse populations and capturing a broader range of real-world practice patterns [4]. At the same time, multicenter trials are not inherently methodologically robust or clinically informative, and their impact depends on careful design, execution, and alignment with meaningful clinical questions.

Understanding what multicenter RCTs have been conducted, and where their strengths and limitations lie, is useful for shaping future research priorities. Despite their potential to influence practice and guideline development, the scope and characteristics of multicenter RCTs in perioperative neuroanesthesia have not been systematically mapped. Specifically, there is limited consolidated information on the scope of perioperative interventions evaluated in multicenter neuroanesthesia trials, the outcomes chosen to assess these interventions, or how trial activity has evolved over time, making it difficult to appraise the overall direction, maturity, and clinical relevance of the existing evidence base.

Given the heterogeneity of interventions, outcome measures, perioperative timing, and trial designs within this field, our objective was not to synthesize comparative treatment effects but rather to systematically map the landscape of existing multicenter randomized evidence, identify recurring themes, and highlight methodological and outcome gaps. A scoping review methodology is particularly well-suited to such questions, as it allows comprehensive characterization of study types, outcome domains, and research trends without restricting inclusion based on intervention homogeneity or prespecifying narrow comparative frameworks [5]. This approach enables clarification of the breadth and maturity of the evidence base and supports the development of a forward-looking research agenda.

Accordingly, we conducted a scoping review to systematically identify and characterize multicenter RCTs evaluating perioperative management strategies in adults undergoing intracranial neurosurgery. The objectives of this review were to: (1) describe the types of perioperative interventions evaluated in multicenter RCTs; (2) characterize outcome selection, including the presence of patient-centered outcomes in neuroanesthesia research; (3) examine temporal and thematic trends in trial activity; and (4) assess key methodological features across studies. By mapping this high-level evidence landscape, we aimed to clarify current research priorities and identify gaps to inform future research and trial design in neuroanesthesia.

## 2. Materials and Methods

### 2.1. Study Design

We conducted a scoping review to identify and characterize multicenter RCTs in neuroanesthesia. The protocol for this study was registered with the International Platform of Registered Systematic Review and Meta-analysis Protocols (registration no. INPLASY202560072).

We deliberately restricted inclusion to multicenter RCTs to prioritize evidence with greater external validity and implementation potential. Multicenter RCTs allow evaluation of perioperative interventions across heterogeneous patient populations and institutional practices while typically providing the statistical power needed for clinically meaningful outcomes. In contrast, single-center trials are often influenced by site-specific protocols and resource availability, constraining generalizability. This approach enabled us to characterize the highest level of translational evidence in neuroanesthesia and to identify relevant research gaps.

The review was reported following the Preferred Reporting Items for Systematic Reviews and Meta-Analyses extension for Scoping Reviews (PRISMA-ScR) checklist [6] (Table S2) and by established frameworks for scoping reviews (Arksey and O’Malley [7], with refinements by Levac et al. [8]).

### 2.2. Eligibility Criteria

Eligible studies were required to meet the following criteria: (1) include adult participants aged 18 years or older undergoing intracranial neurosurgical procedures; (2) employ a RCT design conducted across multiple centers, defined as enrolment from ≥2 distinct recruiting hospitals/clinical sites (as reported by the trial); and (3) evaluate a perioperative management intervention related to anesthetic, hemodynamic, ventilatory, neuromonitoring, neuroprotective, sedation, analgesic, antiemetic, temperature, or other protocolized perioperative strategies. Eligible trials could compare interventions against active comparators, usual care, or placebo. When multicenter status was not explicitly stated in the title or abstract, the full text was reviewed for clarification, including the Methods section, acknowledgments, author affiliations, and any cited protocol or trial registration. Trials were classified as multicenter only if recruitment from ≥2 distinct clinical sites could be confirmed from the published report. If multicenter participation could not be verified, the study was excluded.

We explicitly excluded studies that: (a) evaluated spinal surgery; (b) enrolled pediatric populations; (c) used single-center, non-randomized, observational designs; (d) were published in languages other than English, due to feasibility constraints and limited resources for formal, reliable translation and duplicate data extraction across multiple languages; (e) investigated domains outside the perioperative environment; or (f) were protocols, conference abstracts, or unpublished reports without full trial results, because they typically lack sufficient methodological detail and complete outcome reporting to permit reliable data extraction and risk-of-bias assessment.

### 2.3. Information Sources and Search Strategy

A comprehensive literature search was conducted. The databases MEDLINE (Ovid), PubMed, EMBASE (Ovid), the Cochrane Central Register of Controlled Trials, and Web of Science were searched from inception to 25 June 2025. The combined controlled vocabulary and free-text terms related to “neurosurgery,” “perioperative management,” and “randomized controlled trial” were used. To identify RCTs while minimizing the risk of missing older trials, we did not apply a restrictive validated RCT filter. Instead, we used a sensitive combination of indexed trial terms (e.g., randomized controlled trial/clinical trial publication types/subject headings) and broad free-text trial terms (e.g., random*, blind*) and searched databases from inception. Trial registries were not searched because our aim was to map completed, published multicenter RCTs with extractable methods and outcome data. The MEDLINE search strategy is available in the Supplementary File (Table S1). Reference lists of included studies and relevant systematic reviews were screened manually to identify additional eligible trials.

### 2.4. Study Selection

Titles and abstracts were screened in duplicate independently by two reviewers (ME, AAR) to determine potential eligibility, followed by screening of full-text articles. Discrepancies between reviewers were resolved by a third, independent reviewer (TC). Reasons for exclusion at the full-text stage were recorded.

### 2.5. Extraction

Data were extracted independently by two reviewers (ME, AAR) using a standardized extraction form. Extracted information included publication characteristics, country of origin, year of publication, number of centers, sample size, patient demographics, surgical population, details of the intervention and comparator, timing of intervention within the perioperative period, outcomes measured, and main findings. Data were verified for accuracy, and disagreements were resolved by consensus.

### 2.6. Synthesis of Results

Interventions were grouped into recurring perioperative themes (e.g., anesthetic technique, ventilation/brain relaxation strategy, analgesia, antiemetic prophylaxis, and hemodynamic control) based on our extraction framework to facilitate structured mapping of the literature.

Outcomes were mapped a priori into three domains: (1) patient-centered outcomes, directly reflecting sustained health status, function, or survival (e.g., mortality, functional neurological status, cognitive outcomes, quality of life); (2) patient-important proximal outcomes, defined as clinically meaningful short-term outcomes affecting patient experience or recovery (e.g., pain, postoperative nausea and vomiting, length of stay, postoperative complications); and (3) surrogate/process outcomes, defined as intraoperative or early physiologic or recovery metrics that reflect mechanisms or perioperative processes rather than direct patient function (e.g., hemodynamics, opioid consumption, emergence metrics) [9,10,11,12]. Outcomes were additionally charted by whether they were prespecified as primary endpoints and by timing of assessment (intraoperative, ≤24 h, >24–≤72 h, ≥72 h). Outcome extraction and categorization were informed by the outcome landscape in neuroanesthetic trials and established conceptual frameworks in neuroanesthesia and perioperative outcomes research [9,10,11,12]. This structure reflects recommendations from the neuroanesthesia literature emphasizing the need to distinguish long-term and disease specific patient-centered outcomes such as mortality, neurological function, and cognition that are of great importance to patients, from intermediate clinical endpoints (e.g., pain, postoperative nausea and vomiting, length of stay) and intraoperative or early recovery surrogates (e.g., hemodynamics, opioid consumption, emergence metrics), which are easier to measure but may not reliably translate into meaningful long-term benefit [9,10,11]. When outcomes could reasonably overlap categories or domains, classification was determined based on the highest level of clinical relevance within the hierarchy above (patient-centered > patient-important proximal > surrogate/process). For example, postoperative cognitive testing at hospital discharge was classified as patient-centered, whereas emergence metrics or intraoperative cerebral physiology were classified as surrogate outcomes. Postoperative complications were categorized as patient-important proximal when reported complications were short-term and did not include sustained functional impact measures. Outcome categorization was performed independently by two reviewers (ME, AAR) using this predefined framework. Disagreements regarding domain classification were resolved through discussion and, when necessary, adjudication by a third reviewer (TC).

Risk of bias was assessed for all included trials using the Cochrane Risk of Bias 2 (RoB 2) tool for RCTs [13]. Two reviewers (ME, AAR) independently evaluated each study across the predefined domains, including the randomization process, deviations from intended interventions, missing outcome data, measurement of outcomes, and selection of the reported result. Discrepancies were resolved through discussion and, when necessary, consultation with a third reviewer (TC). The purpose of this assessment was to describe the methodological rigor and common sources of bias within the existing body of multicenter trials rather than to exclude studies or to formally grade the certainty of evidence.

## 3. Results

### 3.1. Study Selection and Characteristics

**Figure 1:**
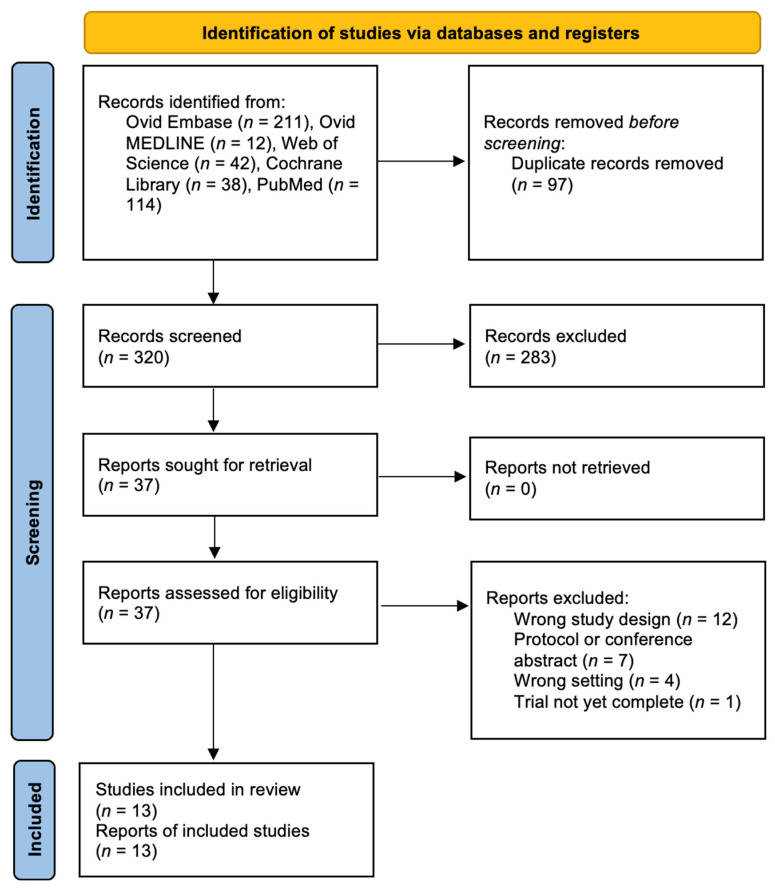
PRISMA Flowchart of Study Selection.

A total of 417 studies were identified by the search, of which 320 remained after removal of duplicates. Thirty-seven studies underwent full-text review and 24 of them were excluded for the following reasons: incorrect study design (*n* = 12), most commonly non-randomized studies or single-center trials that did not meet our multicenter RCT inclusion criteria; protocol-only publications or conference abstracts without full trial reports (*n* = 7); wrong clinical setting (e.g., spinal surgery, traumatic brain injury management, or non-operative neurocritical care) (*n* = 4); and incomplete or ongoing trials without published results (*n* = 1). These exclusion categories are also detailed in the PRISMA flow diagram (Figure 1). Thirteen final studies were included, conducted across nine countries. Two trials were multinational; one conducted in Canada, China, and India [14], and the other conducted in Russia and Italy [15]. The remaining trials were conducted in Türkiye [16], China [17], South Korea [18], Denmark [19], Canada [20], the United States [21,22], and Italy [23,24,25,26]. Years of publication ranged from 1997 to 2025.

Across the 13 trials, 2765 participants were enrolled (range 40–1000 per trial; Table 1). Most trials enrolled adults undergoing elective supratentorial craniotomy for tumor resection (12/13, 92.3%); one large trial enrolled patients undergoing craniotomy for intracranial aneurysm repair (1/13, 7.7%) [22]. Interventions most commonly tested anesthetic maintenance strategies or opioid regimens (7/13, 53.8%), followed by post-craniotomy analgesic strategies (3/13, 23.1%), and single trials evaluating ventilation/brain relaxation [14], antiemetic prophylaxis [18], and intraoperative temperature management [22] (each 1/13, 7.7%).

Interventions were delivered at different perioperative phases: nine trials evaluated intraoperative management (9/13, 69.2%), one evaluated preoperative interventions (1/13, 7.7%), two evaluated postoperative interventions (2/13, 15.4%), and one compared a preoperative to postoperative intervention (Table 1). Follow-up was usually limited to the intraoperative period or the first 24–72 h after surgery; only one trial assessed outcomes at ≥72 h postoperatively, specifically reporting 90-day neurological and functional outcomes [22] (Table 2 and Table S3).

### 3.2. Trial Focus, Outcomes, and Findings

Trial themes and outcomes showed a general temporal pattern. Earlier studies (1997–2005) predominantly compared anesthetic agents and opioid regimens, emphasizing intraoperative physiology and early recovery metrics. From 2008 onward, studies increasingly examined perioperative adjuncts, such as ventilation strategies, regional/topical analgesia, and antiemetic prophylaxis, while outcome selection remained largely short-term.

Early anesthetic technique and opioid regimen trials generally suggested clinical equivalence for common intraoperative and early recovery endpoints. For example, comparisons of TIVA versus inhalational techniques reported similar intraoperative conditions and comparable emergence profiles, with some regimens showing modest differences in recovery speed or early cognitive testing [23,24]. Opioid regimen comparisons (e.g., remifentanil vs. fentanyl-based techniques) reported broadly similar intraoperative stability with minor differences mainly in early neurologic recovery profiles and postoperative analgesic timing [20,21].

Subsequent investigations shifted toward perioperative analgesia and adjunctive measures and demonstrated more consistent short-term symptom benefits. Among three analgesia-focused trials, two evaluated selective scalp block timing and one evaluated preemptive topical lidocaine; these studies reported reductions in early pain scores and/or rescue analgesic requirements in some settings, although effects were modest and not always sustained to 24 h [15,16,17]. Another study reported reduced postoperative nausea and vomiting and lower rescue antiemetic use with ramosetron versus ondansetron after craniotomy [18].

Included trials clustered within a limited number of domains. No multicenter trials were identified that evaluated pharmacologic neuroprotective agents, structured perioperative delirium prevention protocols, multimodal perioperative care pathways, or individualized hemodynamic strategies.

Across all trials, outcome reporting was heavily weighted toward surrogate endpoints. Hemodynamics and opioid use were reported in 7/13 trials (53.8% each), and emergence/recovery metrics in 5/13 (38.5%), whereas patient-centered outcomes were rare: mortality, functional neurological outcome, and cognitive outcome were each reported in only 1/13 trials (7.7%), and no trial measured quality of life (0/13) (Table 2). The single largest trial evaluating intraoperative hypothermia enrolled 1000 patients and found no improvement in neurological outcome at 90 days [22].

### 3.3. Methodological and Reporting Quality

Prospective trial registration was reported in 4/13 trials (30.8%) [16,17,18,23]. Notably, several included trials were conducted prior to the 2005 International Committee of Medical Journal Editors (ICMJE) requirement for prospective trial registration, which likely contributes to the low observed registration rate among earlier studies [27]. However, some trials published after 2005 also did not report publicly available prospective registration [14,15,24,25].

**Figure 2:**
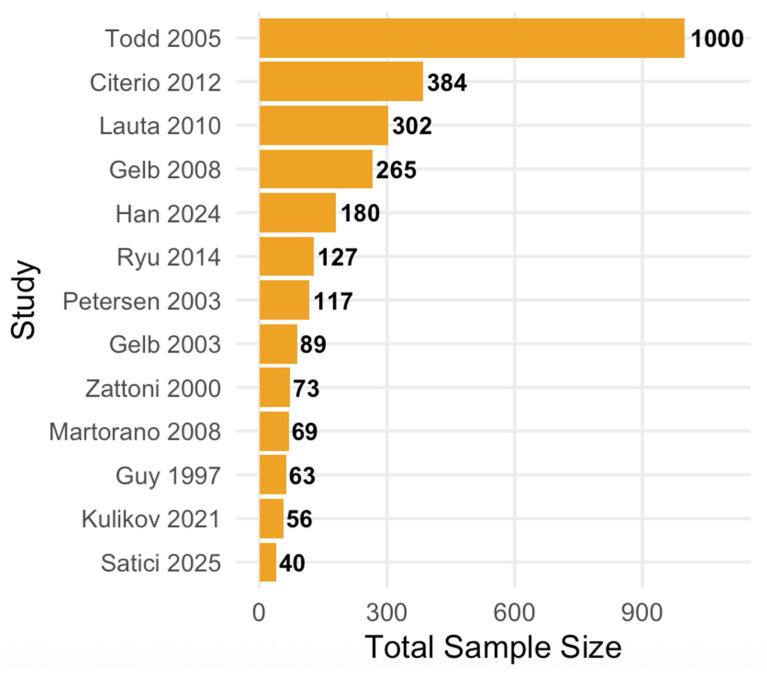
Total Sample Size Distribution of Multicenter Randomized Controlled Trials in Intracranial Neurosurgery.

The two included trials were conducted at two centers [15,16], four studies at three centers [17,19,21,24], three studies at four centers [14,18,26], one study at five centers [20], one trial spanned 14 centers [23], and one trial spanned 30 centers [22]. One study did not specify the number of centers [25]. The sample sizes of the included studies were relatively small. A graph demonstrating the distribution of sample sizes is shown in Figure 2.

Reporting of key methodological safeguards was inconsistent. Random sequence generation and allocation concealment were adequately described in 9/13 (69.2%) and 8/13 (61.5%) trials, respectively (Table 3). Notably, incomplete reporting of these elements was largely confined to studies published prior to 2010. Among trials published after 2010, only one study failed to clearly describe the method of random sequence generation [16], while other key safeguards were all reported. Blinding was described and implemented variably in each study. Five studies were conducted in a double-blind fashion, involving patients, clinicians, and outcome assessors. Four studies implemented partial blinding, limited to the outcome assessor. One trial was open-label, and one did not explicitly provide any information on blinding procedures. Adverse events were explicitly reported in 7/13 trials (53.8%) and were generally limited to transient perioperative events (e.g., hypotension, nausea/vomiting) when reported.

**Figure 3:**
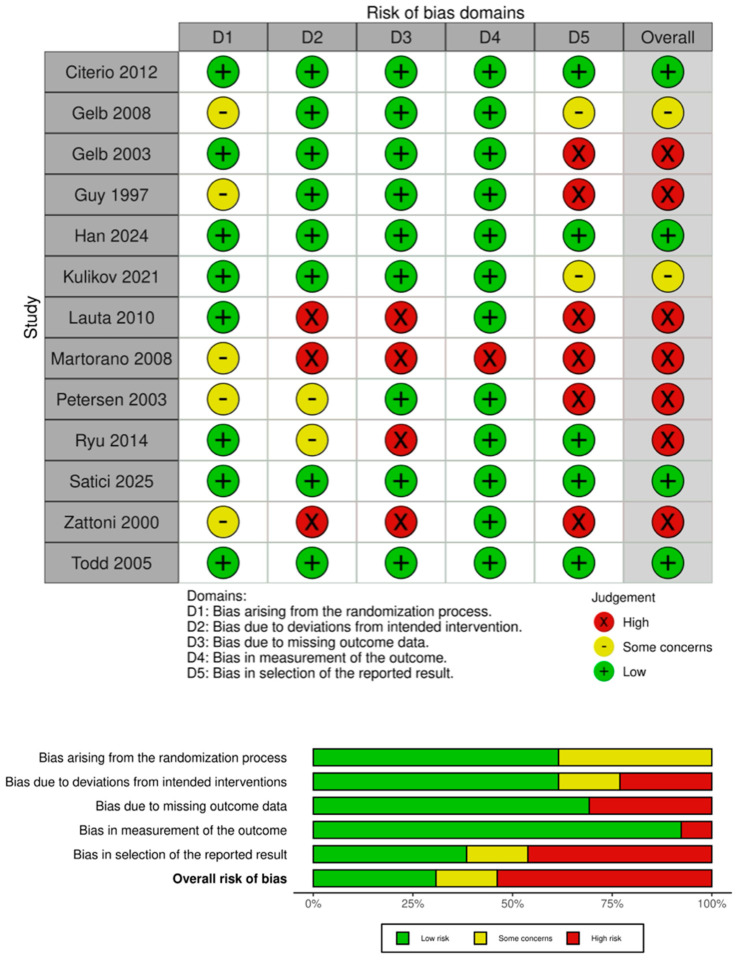
Risk of bias assessment of included multicenter randomized controlled trials. Risk of bias was assessed using the Cochrane Risk of Bias 2 (RoB 2) tool. The upper panel shows the domain-level judgments for each included study (traffic light plot), and the lower panel summarizes the proportion of studies rated as low risk, some concerns, or high risk for each domain and overall. Domains include D1: randomization process; D2: deviations from intended interventions; D3: missing outcome data; D4: measurement of the outcome; and D5: selection of the reported result.

Using the Cochrane RoB 2 tool, the overall risk of bias was judged to be low in four trials, to raise some concerns in two trials, and to be high in seven trials (Figure 3). The most frequent sources of bias were inadequate reporting of the randomization process, lack of prespecified analysis plans, and post-randomization exclusions or non–intention-to-treat analyses.

## 4. Discussion

This scoping review identified 13 multicenter RCTs evaluating perioperative management in intracranial neurosurgery over nearly three decades. All but one trial focused on elective craniotomy patients and primarily examined short-term physiologic or recovery endpoints rather than longer-term neurological outcomes. The scope of interventions studied evolved in tandem with changes in clinical practice and research priorities. Across studies, modern anesthetic techniques (TIVA vs. inhalational) demonstrated broadly comparable intraoperative conditions and early recovery, while later trials shifted toward analgesic adjuncts, antiemetic prophylaxis, and physiologic optimization. However, the collective evidence remains narrow in scope, modest in sample size, and heavily centered on immediate postoperative metrics, consistent with prior observations that neuroanesthesia research has historically emphasized early outcomes at the expense of patient-important longer-term endpoints [10].

A major cross-cutting finding is the mismatch between the breadth of interventions studied and the narrowness of outcome measurement. Across multicenter trials, surrogate and process endpoints (e.g., hemodynamics, opioid consumption, emergence times) predominated, while patient-centered outcomes were uncommon and quality of life was entirely absent (Table 2). This reflects longstanding challenges in neuroanesthesia research: subtle cognitive or neurological changes emerging weeks to months after surgery are difficult and resource-intensive to capture without very large trials [11], leading investigators to default to outcomes readily observable in the operating room or post-anesthesia care unit. The consequence, however, is a literature that risks overemphasizing statistically significant yet clinically minor differences, such as small improvements in emergence time or pain scores, while leaving unresolved whether these interventions meaningfully affect longer-term neurological function, return to baseline activities, or survival. This outcome profile limits interpretability and weakens the connection to clinical decision-making. Future multicenter trials should therefore prespecify outcome hierarchies and adopt standardized core outcome sets for neuroanesthesia, incorporating long-term functional status, cognitive outcomes and delirium, complications, and patient-reported outcomes.

Methodologically, many trials had incomplete reporting of randomization, allocation concealment, and prespecified analyses, particularly among studies published prior to 2010, and more than half were judged high risk of bias. Moreover, very few trials reported prospective registration and publicly available protocols. Although this may partly reflect the inclusion of trials conducted before mandatory registration and reporting policies were widely adopted in the mid-2000s [27], several post-2005 studies also lacked prospective registration, indicating that adherence to evolving transparency standards was inconsistent within the field. Additionally, it was common for trials to enroll on the order of 50–150 patients total, with very few approaching the sample sizes commonly seen in other surgical or anesthetic subspecialties [3,28]. Major complications in elective craniotomy occur at relatively low rates [10], so detecting a reduction in such outcomes would require large sample sizes.

High overall risk of bias in more than half of trials also limits confidence in the reported short-term benefits. There was a predominance of short-term endpoints and recovery metrics with a subjective component in the included trials (e.g., pain scores, nausea/vomiting, and surgeon-rated brain relaxation/field quality, emergence, and Aldrete-based recovery measures). In this context, incomplete blinding and deviations from intended interventions, both observed in a substantial proportion of trials (Figure 3), increase the likelihood that treatment effects are overestimated, particularly for symptom-based or recovery metrics. In addition, risk of bias judgments frequently reflected concerns related to selective reporting and lack of clearly prespecified analysis plans. Given that many trials reported multiple intraoperative and early postoperative endpoints, this raises the possibility that statistically significant findings were preferentially emphasized, further amplifying apparent short-term benefits. By contrast, objective endpoints such as mortality or 90-day neurological outcome are less susceptible to measurement bias; however, these outcomes were rarely assessed in the included multicenter trials, limiting confidence in conclusions beyond the immediate postoperative period.

Nevertheless, the existence of multicenter networks capable of enrolling hundreds to 1000 patients indicates that larger, more definitive neuroanesthesia trials are feasible when the clinical question and outcome set are aligned with patient-important priorities.

Our findings align with broader anesthesiology analyses showing wide practice variation and uneven research coverage across specialties, with a prior comprehensive mapping of multicenter anesthesia trials identifying fewer than 10 multicenter neurosurgical anesthesia RCTs up to 2021 [29]. The current body of multicenter RCT evidence in neuroanesthesia, though invaluable as a foundation, is characterized by moderate methodological quality and limited scope. This means that any conclusions for practice must be drawn with caution. Clinicians and guideline developers should be aware that many routine neuroanesthetic practices still rest on a slim evidence base, and ongoing appraisal of new data is needed as the field evolves.

### 4.1. Strengths and Limitations

This review systematically maps multicenter RCTs in perioperative neuroanesthesia applying rigorous and transparent methodology. The broad inclusion criteria captured the diversity of anesthetic and perioperative interventions relevant to intracranial surgery, providing a structured overview of research activity across three decades and multiple international settings.

Nonetheless, certain limitations must be acknowledged. Because scoping reviews aim to describe rather than synthesize evidence, no formal meta-analysis was undertaken; as such, this review cannot draw conclusions on the comparative efficacy of individual studies. Restriction to English-language publications may have introduced language bias and excluded relevant non-English trials. Given the international scope of neurosurgical and anesthesiology research, it is possible that additional multicenter RCTs conducted in non-English-speaking regions were not captured, which could influence the comprehensiveness of our evidence map and slightly underestimate global trial activity. It is also possible that regional research priorities, perioperative practices, or outcome selections differ across language contexts, meaning that certain intervention domains or patient-centered outcomes may be underrepresented in our analysis. Additionally, despite a comprehensive search strategy, unpublished or ongoing multicenter RCTs may not have been captured, potentially leading to an underestimation of current trial activity and limiting the completeness of the mapped evidence base. Finally, our deliberate focus on multicenter RCTs, while aligned with our objective of mapping higher-level evidence with greater external validity, necessarily excluded high-quality single-center RCTs that may provide valuable mechanistic insights or preliminary data on patient-centered outcomes. As a result, this review should be interpreted as a map of multicenter, practice-shaping evidence rather than a comprehensive synthesis of all randomized neuroanesthesia research.

### 4.2. Research Gaps and Future Directions

Looking forward, our findings point to several concrete priorities for multicenter neuroanesthesia research. First, future trials should prespecify patient-centered primary outcomes and extend follow-up beyond the immediate postoperative window. Based on the gaps identified (Table 2), candidate core outcomes include 30–90 day functional neurological status, delirium and cognitive trajectories, major postoperative complications, mortality, length of stay, and patient-reported quality of life and recovery. These are patient-centered outcomes that have been a recent focus in modern healthcare research [29]. It is important for trials to prioritize such endpoints rather than relying on physiologic or biomarker surrogates alone, given the growing recognition that outcome measures must translate into real functional benefit for neurosurgical patients [30].

Second, the gaps identified in this review (Table 2) map onto several trial priorities that have also been repeatedly highlighted in broader neuroanesthesia research-agenda literature [2]. Building on that overlap, high-yield multicenter RCT “priority questions” in neuroanesthesia that arise include: (1) Can structured perioperative delirium and cognitive protection protocols reduce postoperative delirium and improve longer-term cognitive and functional outcomes after elective craniotomy? Delirium remains common in neurosurgical populations [2], and there is a growing emphasis on delirium, cognition, and consciousness in contemporary anesthesiology research [31], yet no multicenter trials in this review evaluated protocolized prevention strategies. (2) Does a protocolized hemodynamic approach targeting patient-specific cerebral perfusion goals improve new postoperative neurological deficit and functional neurological status compared with usual care? (3) Do bundled perioperative pathways (e.g., ERAS neurosurgical pathways incorporating opioid-sparing analgesia, standardized antiemetic strategy, early mobilization, and discharge criteria) improve quality of recovery and reduce major complications and length of stay? (4) Finally, can monitoring-guided neuroanesthesia strategies (e.g., EEG-based depth strategies and/or cerebral oxygenation monitoring where feasible) reduce delirium and cognitive dysfunction, and improve patient-reported recovery and functional outcomes? Across these questions, future trials should prioritize patient-centered endpoints such as functional neurological status, delirium and cognitive trajectories, major complications, and patient-reported recovery, with follow-up extending beyond the immediate postoperative window whenever feasible.

Third, multicenter trial methods must be strengthened to address recurrent limitations. At minimum, studies should include prospective registration with publicly available protocols, clear allocation concealment, blinded assessment of subjective outcomes, and intention-to-treat analyses. To enhance clinical relevance, investigators should prespecify outcome hierarchies and power studies for meaningful patient-centered endpoints rather than small differences in emergence time or physiologic surrogates. Future trials should also prioritize broader geographic representation, including low- and middle-income settings, as most existing studies were conducted in high-income countries within limited networks. Expanding trial diversity is particularly important in neurosurgical anesthesia, where perioperative strategies may require adaptation across resource contexts to ensure global applicability and equity.

These goals and directions align with national anesthesia research priorities, which emphasize improving patient outcomes and experiences, understanding long-term effects of anesthesia, and optimizing perioperative care, priorities that are directly applicable to neurosurgical populations [32].

## 5. Conclusions

In summary, the current landscape of multicenter RCTs in perioperative neurosurgery is limited but informative. The available evidence suggests that many foundational anesthetic practices are comparable with respect to short-term outcomes, which has practical implications for flexibility in clinical management. At the same time, there is little high-level evidence to inform improvements in long-term neurologic outcomes. The next phase of research should move beyond incremental comparisons toward larger, collaborative trials that examine meaningful patient outcomes and systems-based approaches to perioperative care. Strengthening trial design, ensuring transparent reporting, and adopting standardized measures will be key to building a more reliable evidence base for neuroanesthetic practice.

## Figures and Tables

**Table 1:** Overview of study characteristics of multicenter randomized controlled trials in intracranial neurosurgery.

Citation (Author, Year; Country)	Number of Centers	Type of Surgery	Groups	Sample Size	Age (Mean ± SD)	Sex Distribution, n (%) Male
Satici 2025 [16]; Turkiye	2	Elective craniotomy	Scalp Block	20	48 ± 17	10 (50)
			Control group managed with multimodal analgesia alone	20	51 ± 16	11 (55)
Han 2024 [17]; China	3	Elective craniotomy	Preemptive topical lidocaine 5% white hydrogel plasters	90	48.1 ± 2.7	33 (36.7)
			Control group who received plain hydrogel plasters of the same pattern, size, appearance and material as L5Ps, but free of lidocaine	90	47.0 ± 2.8	40 (44.4)
Kulikov 2021 [15]; Russia and Italy	2	Elective supratentorial craniotomy	Preoperative scalp block combined with incision line infiltration	28	51 ± 17	14 (50)
			Postoperative scalp block combined with incision line infiltration	28	50 ± 12	15 (50)
Ryu 2014 [18]; South Korea	4	Elective craniotomy	4 mg ondansetron intravenously at the time of dural closure	41	49 ± 10.1	14 (34)
			8 mg ondansetron intravenously at the time of dural closure	44	48 ± 8.7	19 (43)
			0.3 mg ramosetron intravenously at the time of dural closure	42	53 ± 9.4	13 (31)
Citerio 2012 [23]; Italy	14	Elective supratentorial craniotomy	Sevoflurane with fentanyl	130	NA	NA
			Sevoflurane with remifentanil	130	NA	NA
			Propofol with remifentanil	124	NA	NA
Lauta 2010 [24]; Italy	3	Elective supratentorial craniotomy	Intravenous anesthesia (propofol/remifentanil-group P)	149	53.1	62 (41.6)
			Inhalation anesthesia (sevoflurane/remifentanil-group S)	153	58.1	75 (49.0)
Gelb 2008 [14]; Canada, China, India	4	Elective supratentorial craniotomy	Hyperventilation followed by normoventilation, propofol infusion	68	47 ± 14	39 (57)
			Hyperventilation followed by normoventilation, isoflurane anesthesia	66	48 ± 17	40 (61)
			Normoventilation followed by hyperventilation, propofol infusion	63	46 ± 13	36 (57)
			Normoventilation followed by hyperventilation, isoflurane anesthesia	68	43 ± 12	39 (57)
Martorano 2008 [25]; Italy	Not specified	Elective supratentorial craniotomy	Sufentanil in combination with propofol	31	52.8 ± 12.8	15 (48.4)
			Remifentanil in combination with propofol	38	56.1 ± 13.5	20 (52.6)
Todd 2005 [22]; USA	30	Craniotomy for intracranial aneurysm	Intraoperative hypothermia (target temperature, 33 °C, with the use of surface cooling techniques)	499	52 ± 12	175 (35)
			Intraoperative normothermia (target temperature, 36.5 °C)	501	51 ± 13	170 (34)
Petersen 2003 [19]; Denmark	3	Elective supratentorial craniotomy	Propofol, Fentanyl	41	55 ± 14	20 (48.7)
			Isoflurane‚ Fentanyl	38	55 ± 10	16 (42.1)
			Sevoflurane‚ Fentanyl	38	53 ± 11	20 (52.6)
Gelb 2003 [20]; Canada	5	Elective supratentorial craniotomy	Thiopental and remifentanil with morphine	44	42 ± 11	24 (54.5)
			Fentanyl with saline	45	45 ± 13	20 (42.6)
Zattoni 2000 [26]; Italy	4	Elective craniotomy	Propofol 1%	37	46.7 ± 8.9	18 (48.6)
			Propofol 2%	36	42.5 ± 13.3	16 (44.4)
Guy 1997 [21]; USA	3	Elective supratentorial craniotomy	Fentanyl	31	49 ± 13	23 (74.2)
			Remifentanil	32	51 ± 13	18 (56.3)

**Table 2:** Outcome reporting across multicenter neuroanesthesia RCTs.

Outcome	Reported	Reported as Primary Outcome	Assessed Intraop	Assessed ≤24 h Postop	Assessed >24 h to ≤72 h Postop	Assessed ≥72 h Postop
**Patient-centered outcomes**						
Mortality	1 (7.7)	0	0	0	0	1 (7.7)
Functional neurological outcomes	1 (7.7)	1 (7.7)	0	0	0	1 (7.7)
Cognitive outcomes	1 (7.7)	1 (7.7)	0	1 (7.7)	0	0
Quality of life	0	0	0	0	0	0
Patient Satisfaction	1 (7.7)	0	0	1 (7.7)	0	0
**Patient-important proximal outcomes**						
Pain	4 (30.8)	4 (30.8)	0	3 (23.1)	2 (15.4)	0
Postoperative complications	2 (15.4)	1 (7.7)	0	1 (7.7)	2 (15.4)	0
Postoperative nausea/vomiting	3 (23.1)	2 (15.4)	0	2 (15.4)	2 (15.4)	0
Length of hospital stay	1 (7.7)	0	0	0	1 (7.7)	0
**Surrogate/process outcomes**						
Cerebral physiology	2 (15.4)	1 (7.7)	2 (15.4)	0	0	0
Opioid consumption and rescue analgesia	7 (53.8)	4 (30.8)	3 (23.1)	6 (46.2)	0	0
Hemodynamics	7 (53.8)	3 (23.1)	7 (53.8)	0	0	0
Emergence/recovery metrics	5 (38.5)	4 (30.8)	2 (15.4)	3 (23.1)	0	0
Surgeon-rated brain relaxation or field quality	2 (15.4)	0	2 (15.4)	0	0	0

The table maps clinically relevant outcomes, frequency of reporting, use as primary endpoints, and timing of assessment. Outcomes are grouped by clinical relevance (patient-centered, patient-important proximal, surrogate/process), with category headings shown in bold. All values reported as n studies (%). Timepoint columns reflect whether studies assessed the outcome within that window. The primary outcome column reflects trials explicitly identifying the outcome as primary.

**Table 3:** Overview of methodology and reporting of multicenter randomized controlled trials in intracranial neurosurgery.

Citation (Author, Year)	Trial Registration and Protocol Reported (ID)	Randomization Method	Allocation Concealment	Blinding	Adverse Events Reported
Satici 2025 [16]	Yes	NR	Sealed opaque envelopes	Double-blind (patients, clinicians, outcome assessors)	Yes
Han 2024 [17]	Yes	Computer-generated random sequence	Sealed opaque envelopes	Double-blind (patients, clinicians, outcome assessors)	Yes
Kulikov 2021 [15]	No	Computer-generated random sequence	Concealed (method not specified)	Double-blind (patients, outcome assessors)	Yes
Ryu 2014 [18]	Yes	Computer-generated random sequence	Sealed opaque envelopes	Double-blind (patients, clinicians, outcome assessors)	Yes
Citerio 2012 [23]	Yes	Centralized randomization via interactive voice response system	Concealed via centralized interactive voice response system	Open label (PROBE design; outcomes assessor blinded)	Yes
Lauta 2010 [24]	No	Block randomization	Sealed opaque envelopes	Partial-blind (outcome assessor)	Yes
Gelb 2008 [14]	No	Computer-generated random sequence	NR	Partial-blind (outcome assessor)	No
Martorano 2008 [25]	No	NR	NR	NR	No
Todd 2005 [22]	No	Block randomization	Sealed opaque envelopes	Partial-blind (outcome assessor)	Yes
Petersen 2003 [19]	No	Block randomization	NR	Partial-blind (outcome assessor)	No
Gelb 2003 [20]	No	Computer-generated random sequence	Pharmacy-prepared identical syringes according to concealed randomization code	Double-blind (patients, clinicians, outcome assessors)	No
Zattoni 2000 [26]	No	NR	NR	Open label	Yes
Guy 1997 [21]	No	NR	NR	Double-blind (patients, clinicians, outcome assessors)	Yes

NR = Not reported in the primary publication.

## Data Availability

No new data were created or analyzed in this study. All data supporting the findings of this scoping review are available within the article and its Supplementary Materials.

## Supplementary Materials

The following supporting information can be downloaded at: https://www.mdpi.com/article/10.3390/jcm15052012/s1, Table S1: MEDLINE Search Strategy; Table S2: Preferred Reporting Items for Systematic reviews and Meta-Analyses extension for Scoping Reviews (PRISMA-ScR) Checklist; Table S3: Overview of study objectives, outcomes, and findings of multicenter randomized controlled trials in intracranial neurosurgery.

## Abbreviations

RCT, randomized controlled trial; PRISMA-ScR, Preferred Reporting Items for Systematic Reviews and Meta-Analyses extension for Scoping Reviews; RoB 2, Risk of Bias 2; TIVA, total intravenous anesthesia; CPP, cerebral perfusion pressure; ICP, intracranial pressure; PONV, postoperative nausea and vomiting; MAP, mean arterial pressure; ASA, American Society of Anesthesiologists; QoL, quality of life.
