# Supplementary material for: Mapping High-Level Evidence in Neuroanesthesia: A Scoping Review of Multicenter Randomized Controlled Trials in Anesthesia for Neurosurgery
Source: J Clin Med. 2026 Mar 6;15(5):2012. doi: 10.3390/jcm15052012 (PMC12986282; doi:10.3390/jcm15052012)
Supplement: Supplementary file 1 [file jcm-15-02012-s001.zip › jcm-4172221-supplementary.pdf]

# Supplementary File

**Table S1:** MEDLINE Search Strategy
Ovid MEDLINE(R) ALL 1946 to June 24, 2025

| # | Searches | Results |
|---|---|---|
| 1 | exp Craniotomy/ | 18855 |
| 2 | exp Neurosurgery/ | 17782 |
| 3 | "craniotom*".ab,ti. | 19203 |
| 4 | "craniectom*".ab,ti. | 6214 |
| 5 | "decompressive craniectom* ".ab,ti. | 2884 |
| 6 | "brain surgery".ab,ti. | 1764 |
| 7 | "intracranial surgery".ab,ti. | 799 |

| | | |
|---|---|---|
| 8 | 1 or 2 or 3 or 4 or 5 or 6 or 7 | 52939 |
| 9 | exp Anesthesia/ or exp "Anesthesia and Analgesia"/ | 261789 |
| 10 | exp Analgesia/ | 51855 |
| 11 | exp Neuroanesthesia/ | 6 |
| 12 | "anesthesia*".ab,ti. | 193791 |
| 13 | "perioperative management".ab,ti. | 9209 |
| 14 | "analgesia*".ab,ti. | 76875 |
| 15 | "intraoperative management".ab,ti. | 1453 |
| 16 | "postoperative management".ab,ti. | 7373 |
| 17 | "preoperative management".ab,ti. | 1463 |
| 18 | "anesthetic technique".ab,ti. | 2567 |

| | |
|---|---|
| 19 neuroanesthesia.ab,ti. | 413 |
| 20 "postoperative care".ab,ti. | 9264 |
| 21 "perioperative care".ab,ti. | 6021 |
| 22 "preoperative care".ab,ti. | 721 |
| 23 9 or 10 or 11 or 12 or 13 or 14 or 15 or 16 or 17 or 18 or 19 or 20 or 21 or 22 | 427211 |
| 24 exp Randomized Controlled Trial/ | 643493 |
| 25 "level I evidence".ab,ti. | 915 |
| 26 "high-level evidence".ab,ti. | 2100 |
| 27 "randomized controlled trial".ab,ti. | 128403 |
| 28 "RCT".ab,ti. | 38565 |
| 29 "randomized clinical trial".ab,ti. | 46540 |

| | |
|---|---|
| 30 "cluster randomized trial".ab,ti. | 4289 |
| 31 "controlled clinical trial".ab,ti. | 21885 |
| 32 "clinical trial".ab,ti. | 226482 |
| 33 "Double-Blind Method".ab,ti. | 482 |
| 34 "Single-Blind Method".ab,ti. | 93 |
| 35 "random*".ab,ti. | 1642239 |
| 36 24 or 25 or 26 or 27 or 28 or 29 or 30 or 31 or 32 or 33 or 34 or 35 | 1882304 |
| 37 Multicenter Studies as Topic/ | 26044 |
| 38 "multicenter trial* ".ab,ti. | 11072 |
| 39 "multicenter stud* ".ab,ti. | 45698 |
| 40 "multicenter*".ab,ti. | 175164 |

| | |
|---|---|
| 41 "multicentre*".ab,ti. | 54621 |
| 42 "multi-center* ".ab,ti. | 20642 |
| 43 "multi-centre".ab,ti. | 10818 |
| 44 "multi-site".ab,ti. | 8372 |
| 45 Multicentric.ab,ti. | 19737 |
| 46 37 or 38 or 39 or 40 or 41 or 42 or 43 or 44 or 45 | 296983 |
| 47 8 and 23 and 36 and 46 | 24 |

**Table S2:** Preferred Reporting Items for Systematic reviews and Meta-Analyses extension for Scoping Reviews (PRISMA-ScR) Checklist

| SECTION | ITEM | PRISMA-ScR CHECKLIST ITEM | REPORTED ON PAGE # |
|---|---|---|---|
| **TITLE** | | | |
| Title | 1 | Identify the report as a scoping review. | 1 |

| | | | |
|---|---|---|---|
| **ABSTRACT** | | | |
| Structured summary | 2 | Provide a structured summary that includes (as applicable): background, objectives, eligibility criteria, sources of evidence, charting methods, results, and conclusions that relate to the review questions and objectives. | 2-3 |
| **INTRODUCTION** | | | |
| Rationale | 3 | Describe the rationale for the review in the context of what is already known. Explain why the review questions/objectives lend themselves to a scoping review approach. | 4-5 |
| Objectives | 4 | Provide an explicit statement of the questions and objectives being addressed with reference to their key elements (e.g., population or participants, concepts, and context) or other relevant key elements used to conceptualize the review questions and/or objectives. | 5 |
| **METHODS** | | | |
| Protocol and registration | 5 | Indicate whether a review protocol exists; state if and where it can be accessed (e.g., a Web address); and if available, provide registration information, including the registration number. | 5 |
| Eligibility criteria | 6 | Specify characteristics of the sources of evidence used as eligibility criteria (e.g., years considered, language, and publication status), and provide a rationale. | 6-7 |
| Information sources* | 7 | Describe all information sources in the search (e.g., databases with dates of coverage and contact with authors to identify additional sources), as well as the date the most recent search was executed. | 7 |
| Search | 8 | Present the full electronic search strategy for at least 1 database, including any limits used, such that it could be repeated. | Appendix |
| Selection of sources of evidence† | 9 | State the process for selecting sources of evidence (i.e., screening and eligibility) included in the scoping review. | 7 |

| | | | |
|---|---|---|---|
| Data charting process‡ | 10 | Describe the methods of charting data from the included sources of evidence (e.g., calibrated forms or forms that have been tested by the team before their use, and whether data charting was done independently or in duplicate) and any processes for obtaining and confirming data from investigators. | 7-8 |
| Data items | 11 | List and define all variables for which data were sought and any assumptions and simplifications made. | 7-8 |
| Critical appraisal of individual sources of evidence§ | 12 | If done, provide a rationale for conducting a critical appraisal of included sources of evidence; describe the methods used and how this information was used in any data synthesis (if appropriate). | 9 |
| Synthesis of results | 13 | Describe the methods of handling and summarizing the data that were charted. | 8 |
| **RESULTS** | | | |
| Selection of sources of evidence | 14 | Give numbers of sources of evidence screened, assessed for eligibility, and included in the review, with reasons for exclusions at each stage, ideally using a flow diagram. | 9 |
| Characteristics of sources of evidence | 15 | For each source of evidence, present characteristics for which data were charted and provide the citations. | 9-10 |
| Critical appraisal within sources of evidence | 16 | If done, present data on critical appraisal of included sources of evidence (see item 12). | Figure 3 |
| Results of individual sources of evidence | 17 | For each included source of evidence, present the relevant data that were charted that relate to the review questions and objectives. | Tables 1-3 |
| Synthesis of results | 18 | Summarize and/or present the charting results as they relate to the review questions and objectives. | 10-22 |
| **DISCUSSION** | | | |
| Summary of evidence | 19 | Summarize the main results (including an overview of concepts, themes, and types of evidence available), link to the review questions and objectives, and consider the relevance to key groups. | 22 |
| Limitations | 20 | Discuss the limitations of the scoping review process. | 24 |
| Conclusions | 21 | Provide a general interpretation of the results with respect to the review questions and objectives, as well as potential implications and/or next steps. | 22-26 |
| **FUNDING** | | | |

| | | | |
|---|---|---|---|
| Funding | 22 | Describe sources of funding for the included sources of evidence, as well as sources of funding for the scoping review. Describe the role of the funders of the scoping review. | 27 |

**Table S3:** Overview of study objectives, outcomes, and findings of multicenter randomized controlled trials in intracranial neurosurgery

| Citation (author, year) | Population | Timing of Intervention (perioperative phase) | Objective | Primary Outcomes Measured | Secondary Outcomes Measured | Follow-Up Duration/time-points | Key Findings |
|---|---|---|---|---|---|---|---|
| **Satici 2025** | ASA status I-III adult patients aged 19–65 y | Postoperatively | To evaluate the effectiveness of the scalp block in reducing postoperative pain in craniotomy patients, assessed using the numerical rating scale. | NRS pain scores at 0, 2, 4, 6, 8, 12, and 24 hours postoperatively | The total amount of tramadol administered as rescue analgesia, the time elapsed before the first dose of rescue analgesics, the requirement for antiemetic medication, the incidence of postoperative nausea and vomiting, and patient satisfaction levels. | 0h, 2h, 4h, 6h, 8h, 12h, 24h | The scalp block group demonstrated significantly lower NRS scores than controls at all measured time points. Tramadol consumption was significantly reduced in the scalp block group compared to controls. |
| **Kulikov 2021** | Adult patients aged 18 y or older | Preoperatively | To determine the optimal timing for selective scalp block in patients undergoing general anesthesia for supratentorial craniotomy. | VAS score 24 hours after surgery | VAS score 2, 6, and 12 hours after surgery; time of patient's first request for rescue analgesia; intraoperative consumption of anesthetics and opioids; awakening time (time between interruption of the administration of propofol or sevoflurane to extubation); and perioperative complications (eg, episodes of hemodynamic instability). | 2h, 6h, 12h, 24h | There was no difference in severity of pain at 24, 12, 6, and 2 hours after surgery between the 2 study groups, but the amount of fentanyl administered intraoperatively was lower in patients assigned to the preoperative scalp block group |

| Citation (author, year) | Population | Timing of Intervention (perioperative phase) | Objective | Primary Outcomes Measured | Secondary Outcomes Measured | Follow-Up Duration/time-points | Key Findings |
|---|---|---|---|---|---|---|---|
| **Han 2024** | Adult patients aged 18 y or older | Preoperatively and postoperatively | To evaluate the efficacy profile of preemptive application of a topical 5% lidocaine patch in alleviating post-craniotomy pain. | VAS score 24 hours after surgery | Intra-operative analgesics consumption, pain intensity, cumulative rescue analgesics consumption, sleeping scores, adverse effects such as skin reactions | 1h, 4h, 6h, 12h, 48h, 72h | There were no statistically significant differences in the VAS scores at 24 h after craniotomy. Statistically significant differences were found in VAS scores in male patients. |
| **Ryu 2014** | ASA status I-II adult patients aged 19-65 y | Postoperatively | To evaluate the efficacy of prophylactic ramosetron in preventing PONV compared with ondansetron after elective craniotomy in adult patients | PONV, the need for rescue antiemetics, pain score, patient-controlled analgesia consumption, and adverse events | NA | 48h | Ramosetron 0.3 mg lowered the rates of nausea and vomiting and reduced the need for additional antiemetics after craniotomy, showing greater effectiveness than ondansetron 4 or 8 mg. |
| **Citerio 2012** | Adults patients aged 18-75 y | Intraoperative | To test equivalence of inhalational and intravenous anesthesia maintenance techniques in the postoperative recovery of patients undergoing elective supratentorial surgery | The time to achieve an Aldrete post-anesthesia score of at least 9 after tracheal extubation | Hemodynamic parameters, quality of the surgical field, perioperative neuroendocrine stress responses and routine postoperative assessments. | Intraoperative | Equivalence was shown for inhalational and intravenous maintenance anesthesia in times to reach an Aldrete score of at least 9 after tracheal extubation. Hemodynamic variables, the quality of surgical field and postoperative assessments were also similar. Perioperative endocrine stress responses were significantly blunted with propofol remifentanil and higher analgesic requirements were recorded in the remifentanil groups. |

| Citation (author, year) | Population | Timing of Intervention (perioperative phase) | Objective | Primary Outcomes Measured | Secondary Outcomes Measured | Follow-Up Duration/time-points | Key Findings |
|---|---|---|---|---|---|---|---|
| **Lauta 2010** | ASA status I-III adult patients 18 - 75 y | Intraoperative | To test the hypothesis that inhalation anesthesia (sevoflurane/remifentanil) reduces emergence time by 5 minutes compared with intravenous anesthesia (propofol/remifentanil) in patients undergoing neurosurgery for supratentorial neoplasms. | Time to reach an Aldrete test score of more than equal to 10 | Times to eyes opening and extubation, adverse events, intraoperative hemodynamics, brain relaxation score, opioid consumption, and diuresis. | First 3 postoperative hours; Aldrete test at 5, 10, and 15 minutes after extubation and then every 15 minutes for 3 hours | No significant differences were found between the two groups in primary outcomes. Sevoflurane/remifentanil neuroanesthesia is not superior to propofol/remifentanil in time to reach an Aldrete score > 10 |
| **Gelb 2008** | ASA status I-III adult patients 18 - 75 y | Intraoperative | To evaluate the efficacy of moderate hyperventilation in patients undergoing craniotomy for excision of supratentorial brain tumors during isoflurane or propofol anesthesia | Surgeon-assessed brain bulk | Subdural ICP | Intraoperative | In patients with supratentorial brain tumours, intraoperative hyperventilation improves surgeon-assessed brain bulk which was associated with a decrease in ICP. |

| Citation (author, year) | Population | Timing of Intervention (perioperative phase) | Objective | Primary Outcomes Measured | Secondary Outcomes Measured | Follow-Up Duration/time-points | Key Findings |
|---|---|---|---|---|---|---|---|
| **Martorano 2008** | Adult patients 18 - 75 y | Intraoperative | To test the hypothesis that sufentanil, on agreement with its pharmacokinetic and pharmacodynamic profile, when used in neurosurgical procedures, provides the same hemodynamic stability and rapid recovery compared with remifentanil with better postoperative characteristics. | Intraoperative hemodynamic effects, recovery profiles, and postoperative characteristics | NA | Intraoperatively and 15, 45, and 180 min after emergence from anesthesia. | There were no significant differences between the groups in the duration of surgery and anesthesia, mean arterial pressure, heart rate, time to eye opening or extubation. The incidence of vomiting, respiratory depression and shivering was similar in both groups. Postoperative pain requiring supplemental analgesics was significantly lower in the sufentanil group. Although there were no significant differences between the groups in postoperative behavioral examinations by Rancho Los Amigos Test, patients anesthetized with sufentanil had significantly better Short Orientation-Memory-Concentration Test values at 15 and 180 min postoperatively. |

| Citation (author, year) | Population | Timing of Intervention (perioperative phase) | Objective | Primary Outcomes Measured | Secondary Outcomes Measured | Follow-Up Duration/time-points | Key Findings |
|---|---|---|---|---|---|---|---|
| **Todd 2005** | ASA status II-III adult patients aged 18 y or older | Intraoperative | To determine whether intraoperative cooling during open craniotomy would improve the outcome among patients with acute aneurysmal subarachnoid hemorrhage. | A score of 1 on the Glasgow Outcome Scale (indicating mild or no disability) | The Rankin scale, the Barthel index, the National Institutes of Health Stroke Scale, and a battery of neuropsychological examinations. | 90 days postoperatively | There were no significant differences between the group assigned to intraoperative hypothermia and the group assigned to normothermia in the duration of stay in the intensive care unit, the total length of hospitalization, the rates of death at follow-up, or the destination at discharge. Intraoperative hypothermia did not improve the neurologic outcome of patients. |
| **Petersen 2003** | Adult patients 18-70 y | Intraoperative | To investigate differences in subdural intracranial pressure (ICP) before and during hyperventilation and to study the incidence of cerebral swelling after opening of the dura, as well as effects on CPP, AVDO2, CO2 reactivity, and dural tension. | Subdural ICP, dural tension, cerebral swelling | CPP, AVDO2, SjvO2, $CO_2$ reactivity | Intraoperative, before and during hyperventilation, after opening of dura | ICP significantly lower and CPP higher in propofol group compared with isoflurane and sevoflurane; cerebral swelling less in propofol group |
| **Gelb 2003** | ASA status I-III adult patients 18 - 65 y | Intraoperative | To compare the recovery profiles, efficacy and safety of remifentanil with morphine transitional analgesia and fentanyl in patients undergoing elective craniotomy for supratentorial mass lesions. | The time from the end of surgery to extubation | responses to surgical stimuli, vital signs, need for rescue medications and recovery times for verbal response and ability to follow commands | Early postoperative, up to 8 hours postoperatively | Patients given fentanyl had higher blood pressure during induction and intubation. Both groups were extubated after about 8 minutes, but recovery was more consistent with remifentanil. Those receiving remifentanil regained normal neurological function sooner and were rated as more alert by staff, though they needed pain medication sooner after surgery. |

| Citation (author, year) | Population | Timing of Intervention (perioperative phase) | Objective | Primary Outcomes Measured | Secondary Outcomes Measured | Follow-Up Duration/time-points | Key Findings |
|---|---|---|---|---|---|---|---|
| **Zattoni 2000** | ASA status I-II adult patients 18 - 60 y | Intraoperative | To investigate whether the 1% and 2% formulations of propofol are equivalent in terms of clinical pharmacodynamic parameters and to compare the effects of the two formulations on plasma cholesterol and triglyceride concentrations in patients undergoing elective craniotomy. | Induction time, induction dose | Recovery time, hemodynamic variables, plasma cholesterol and triglyceride concentration, tolerability | Intraoperative and 1, 2 and 24 hours postoperatively | Propofol 1% and propofol 2% are pharmacodynamically equivalent. Both formulations were similar regarding administration rates, recovery times, hemodynamic variables and tolerability. Plasma triglyceride levels were lower in the propofol 2% group compared with the propofol 1% group, and significantly lower from 1 to 4 hours after induction. |
| **Guy 1997** | ASA status II-III adult patients aged 18 y or older | Intraoperative | To compare the safety and efficacy of remifentanil and fentanyl in patients requiring craniotomy for supratentorial space-occupying lesions. | Hemodynamics, time to recovery, analgesic requirements, nausea and vomiting, and neurological examinations | NA | Intraoperative and 1 and 24 hours postoperatively | Blood pressure was similar at the start of anesthesia but rose higher in patients given fentanyl after intubation. Intracranial and cerebral pressures were comparable between groups, though fentanyl patients required more isoflurane. Both groups were extubated in similar times, but only fentanyl patients needed naloxone. Those who received remifentanil required pain medication sooner, while postoperative blood pressure was higher in that group. Rates of nausea and vomiting were alike. |
